# Describing Events: Changes in Eye Movements and Language Production Due to Visual and Conceptual Properties of Scenes

**DOI:** 10.3389/fpsyg.2019.00835

**Published:** 2019-04-17

**Authors:** Yulia Esaulova, Martina Penke, Sarah Dolscheid

**Affiliations:** Department of Special Education and Rehabilitation, University of Cologne, Cologne, Germany

**Keywords:** sentence production, visual attention, cueing, animacy, left-to-right preferences, active and passive voice, thematic roles, event scenes

## Abstract

How can a visual environment shape our utterances? A variety of visual and conceptual factors appear to affect sentence production, such as the visual cueing of patients or agents, their position relative to one another, and their animacy. These factors have previously been studied in isolation, leaving the question about their interplay open. The present study brings them together to examine systematic variations in eye movements, speech initiation and voice selection in descriptions of visual scenes. A sample of 44 native speakers of German were asked to describe depicted event scenes presented on a computer screen, while both their utterances and eye movements were recorded. Participants were instructed to produce one-sentence descriptions. The pictures depicted scenes with animate agents and either animate or inanimate patients who were situated to the right or to the left of agents. Half of the patients were preceded by a visual cue – a small circle appearing for 60 ms on a blank screen in the place of patients. The results show that scenes with left- rather than right-positioned patients lead to longer speech onset times, a higher probability of passive sentences and looks toward the patient. In addition, scenes with animate patients received more looks and elicited more passive utterances than scenes with inanimate patients. Visual cueing did not produce significant changes in speech, even though there were more looks to cued vs. non-cued referents, demonstrating that cueing only impacted initial scene scanning patterns but not speech. Our findings demonstrate that when examined together rather than separately, visual and conceptual factors of event scenes influence different aspects of behavior. In comparison to cueing that only affected eye movements, patient animacy also acted on the syntactic realization of utterances, whereas patient position in addition altered their onset. In terms of time course, visual influences are rather short-lived, while conceptual factors have long-lasting effects.

## Introduction

When people produce an utterance, they have a number of different linguistic options at their disposal. For instance, they could describe an event with a man kissing a woman by means of a simple transitive clause such as “The man is kissing the woman,” with a cleft-construction “It is the man who is kissing the woman” or with a passive sentence “The woman is being kissed by the man,” just to name some of the options feasible in English. Crucially, a number of factors appear to affect the way speakers choose a particular syntactic structure. One of the most well documented factors influencing the choice of syntactic constructions is the animacy status of a referent (e.g., Bock et al., 1992; McDonald et al., 1993; van Nice and Dietrich, 2003; Tanaka et al., 2011). In the example above, both referents (the woman and the man) are animate. However, numerous studies have shown that in case of an animate and an inanimate referent, animates are more likely to be realized as the subject of an utterance (e.g., McDonald et al., 1993; van Nice and Dietrich, 2003). For instance, in a sentence recall paradigm, McDonald et al. (1993) asked English speaking participants to reproduce sentences involving an animate and an inanimate entity that they had heard (e.g., The music soothed the child). The authors found that participants were more likely to erroneously remember animate referents as subjects compared to inanimates, even if this resulted in the production of passive constructions (e.g., The child was soothed by the music). Similarly, Tanaka et al. (2011) showed that speakers of Japanese – like English speakers – were more likely to erroneously recall animate referents as sentence subjects, confirming an increase in patient-first structures (i.e., passives) when patients were animate. They also found that Japanese speakers were more likely to assign animate referents earlier positions in the sentence than inanimate referents, suggesting that animacy affected both syntactic structure (i.e., the rate of passivizations) and word order (see Tanaka et al., 2011). Beyond sentence recall paradigms, animacy has also been shown to affect the choice of syntactic structure when participants had to describe visual events (e.g., van Nice and Dietrich, 2003; van de Velde et al., 2014). For instance, van Nice and Dietrich (2003) found that German-speaking participants produced more passive constructions when the agent of a transitive action was inanimate rather than animate. That is, a passive construction was more likely to be produced when an inanimate entity exerted an action (e.g., a wheelchair pushing a pig) than when the action was performed by an animate referent (e.g., a bear). Thus, the number of passives was higher for the former event (“the pig is pushed by the wheelchair”) compared to the latter (“the bear pushes the pig”; see van Nice and Dietrich, 2003). A similar effect was obtained when the animacy status of the agent was held constant but animacy of the patient varied (e.g., a suitcase vs. a pig being pushed by a bear). German speakers produced more passive constructions when they had to describe pictures in which the patient of an action was animate (“The pig is pushed by the bear”) compared to inanimate patients (“The bear pushes the suitcase”; van Nice and Dietrich, 2003). The increase of passivizations for animate patients was also confirmed in speakers of Dutch (van de Velde et al., 2014), supporting the importance of animacy for speakers’ structural choices in sentence production.

In addition to the conceptual factor of referent animacy, there is evidence that other factors, too, can exert similar effects on sentence formulation. For instance, drawing the attention of a speaker to a referent by means of a visual cue can likewise affect participants’ structural choices (e.g., Tomlin, 1995, 1997; Gleitman et al., 2007; Myachykov et al., 2011, 2012). In his seminal “fish film,” for instance, Tomlin (1995, 1997) presented English-speaking participants with video clips depicting two fish approaching each other from the left and the right side of the screen. Each scene ended with one of the fish swallowing the other. Visual attention was manipulated by an explicit arrow either pointing to the agent or to the patient fish on a given trial. Participants were more likely to describe the scene with a passive construction (The blue fish is being eaten by the red fish) when the patient fish had been the center of visual attention than when the agent fish had been cued. These results suggest that attention orienting can affect structural choices – similar to the conceptual factor animacy. That is, a visually salient referent is more likely to be realized as the more prominent subject of the utterance even if this requires the production of a more marked passive construction. While Tomlin’s task was criticized for a number of reasons (e.g., Gleitman et al., 2007), other studies replicated Tomlin’s original findings in more carefully controlled set-ups (Gleitman et al., 2007; Myachykov et al., 2011, 2012). For instance, Gleitman et al. (2007) presented participants with pictures of simple transitive events depicting two characters (agent and patient; e.g., a man kicking a boy). Before watching the events, participants’ attention was manipulated by means of a subliminal visual cue that either drew the speakers’ attention to the agent or to the patient of the event. When the cue directed participants’ attention to the patient location, speakers were more likely to produce passive voice sentences compared to cued agents, thus confirming Tomlin’s original findings.

While these studies suggest that visual attention may exert an effect on participants’ structural choices that is comparable to the one demonstrated for animacy, so far both factors have been mainly studied in isolation (for one exception albeit with a different focus see van de Velde et al., 2014). That is, studies investigating effects of animacy on syntactic choice tended to ignore the visual saliency of a referent (e.g., McDonald et al., 1993; van Nice and Dietrich, 2003; Tanaka et al., 2011). Conversely, the majority of studies demonstrating effects of visual attention on sentence formulation exclusively included referents matched for animacy (e.g., Gleitman et al., 2007; Myachykov et al., 2011, 2012). As a consequence, whether or not the two factors really affect sentence formulation in similar ways is still unknown. To fill this gap, the present study sought to simultaneously examine effects of referent animacy and visual saliency (i.e., attentional cueing) on speakers’ sentence production in an eye-tracking study. Do both factors have a similar effect on sentence formulation or is one more important than the other? It is possible that conceptual properties of a referent such as animacy are more relevant and exert stronger effects on sentence formulation than visual cues. While evidence from sentence production is missing so far, some demonstration in favor of this proposal comes from studies investigating visual scene perception. That is, participants’ looking behavior in a free-viewing scene description task was affected more by conceptual aspects than by visually salient objects when meaning maps representing the spatial distribution of semantic features and saliency maps representing the distribution of image features were compared directly (e.g., Henderson et al., 2018). In contrast, a study by Rissman et al. (2018) focusing on participants’ written descriptions of transitive events seems to indicate a reversed effect. In particular, these authors demonstrated that conceptual properties such as animacy of an agent can be overridden when the agent is visually backgrounded (i.e., by only presenting the agent’s torso or hand; see Rissman et al., 2018). Asked to describe visual scenes of transitive events in which an animate agent performed an action on an inanimate entity, participants used more passive constructions when the animate agent was perceptually minimized than when not (Rissman et al., 2018). This was true despite the fact that participants judged both full and partial agents to have the same degree of animacy, suggesting that visual saliency may have the potential to override an effect as important as the animacy of the agent. Crucially, however, since participants in the study by Rissman et al. (2018) were asked to type their responses, it remains unclear which role both factors (animacy and visual saliency) play during spoken language production – a question addressed in the present study. Unlike previous studies, we not only focused on participants’ structural choices (i.e., the rate of active vs. passive sentences) but also included analyses of speech onset times, as well as participants’ looking behavior during the course of an utterance planning and production (by means of eye tracking). This way, the present study offers a first comprehensive approach to how the two different factors (animacy and visual saliency) affect sentence production.

In addition to examining the effects of referent animacy and visual saliency, we also focused on another factor, which has not yet been investigated in sentence production despite its attested relevance for language comprehension. This factor concerns the relative positioning of referents in transitive events, i.e., whether a patient of an action is depicted to the right or to the left of an agent in a visual scene. The positioning of referents has been shown to persistently influence a number of behavioral responses, such as drawings (Maass et al., 2014), aesthetic judgments (McLaughlin and Murphy, 1994; Maass et al., 2007), and spatial memory recalls (Maass et al., 2014). For instance, when participants listened to simple transitive sentences like “The circle hits the square” and then subsequently had to draw the event, they located agents to the left of the patient rather than to the right (Chatterjee et al., 1999). A similar left-to-right preference was observed for speakers of Italian (e.g., Maass and Russo, 2003), as well as German speakers (Dobel et al., 2007). Despite these visual preferences for referents in transitive events, the effect of visual positioning has so far evaded the focus of language production studies. Thus, most of the studies examining sentence production during scene descriptions have counterbalanced this factor rather than systematically exploring its effect (e.g., Myachykov et al., 2018a). However, some authors observed that speakers of English produced more active sentences when describing pictures in which the agent was located on the left of the patient than when the agent was located on the right, indicating a preferred left-to-right mapping of the depicted referents (e.g., Bock, 1986, also see Hartsuiker and Kolk, 1998). Conversely, more passives were produced when the patient was presented to the left of the agent. Taken together, these findings indicate the possibility that the visual arrangement of a transitive event might likewise affect sentence formulation, similar to factors such as animacy and visual saliency of a referent. To test this assumption, we explicitly added the positioning of visual referents (i.e., agent on the left of a patient vs. agent on the right of a patient) as an experimental factor.

### Overview of the Present Study

The present study addressed the question of the relative importance of visual and conceptual factors for language production in the context of a scene description task. The considered factors thus included the conceptual factor animacy (animate vs. inanimate patient) and two visual factors: cueing (cue vs. no cue on patients) and positioning of patients relative to agents (left vs. right). The assessed behavioral aspects comprised language production (the type of utterances produced and their onset times) and visual behavior (eye movement patterns monitored over the course of an utterance). This combination of behavioral measures should provide complementary information about the influence of the manipulated factors on sentence planning. The methodological approach of our study thus goes beyond the analysis of utterance types, which has so far been the focus of the existing studies on language production using picture-description paradigms. The predictions made in regards of each of the manipulated factors are described below.

If animate entities are indeed more likely to be realized as subjects and take sentence-initial positions, as claimed by previous research (e.g., MacDonald et al., 1994; van Nice and Dietrich, 2003), we should observe a higher number of passive voice descriptions of scenes with animate rather than inanimate patients. Moreover, the presence of an animate patient in addition to an animate agent may result in a competition for the subject position between the two and lead to later utterance onsets than when only the agent is animate. In terms of visual behavior, if animate patients are perceived as conceptually more relevant than inanimate patients, this should be reflected in earlier and longer looks to them compared to their inanimate counterparts.

Drawing visual attention to patients via cueing should first of all affect the visual behavior, so that patients should be fixated before agents. If attention orienting also affects structural choices in sentence production (as shown in, e.g., Tomlin, 1995; Myachykov et al., 2012), then we should observe more passive descriptions of scenes following cueing of the patient than scenes where no cueing occurred. Deviation from the preferred active voice structure might require more processing time. We should, hence, also observe longer speech onset times in the patient cueing condition compared to the no cueing condition.

The positioning of elements in scenes has not yet been investigated in speech-production tasks. Generalizing the left-agent preference reported for language comprehension tasks to language production (e.g., Maass and Russo, 2003), we would expect patients positioned to the left of agents to elicit longer speech onset times, more passive voice utterances, as well as earlier and longer looks to them compared to patients positioned to the right of agents.

Since so far no study compared these conceptual and visual factors directly within one design, we cannot derive any predictions about possible interactions or the weight of factors relative to one another. On the one hand, it is possible that the factors may impact participants’ behavior in a cumulative manner, simply adding up – in which case we should observe main effects but no interactions. On the other hand, it is also possible that the effect of one factor may depend on the effect of the other resulting in an interaction.

Additionally, since we not only study structural choices but also inspect participants’ speech onset times as well as visual gaze patterns, it is possible that these measures are influenced to a similar degree by the tested experimental factors. Alternatively, the different aspects of verbal and visual behavior may be affected differently by the factors under study.

## Materials and Methods

### Participants

Forty-four students at the University of Cologne (36 female and 8 male; mean age 23.43 years, *SD* = 3.01) were offered a monetary compensation or a course credit for their participation in the experiment. All of them were native speakers of German who did not report any attention or language-related medical condition and had normal or corrected to normal vision.

### Materials

#### Experimental Stimuli

**FIGURE 1:**
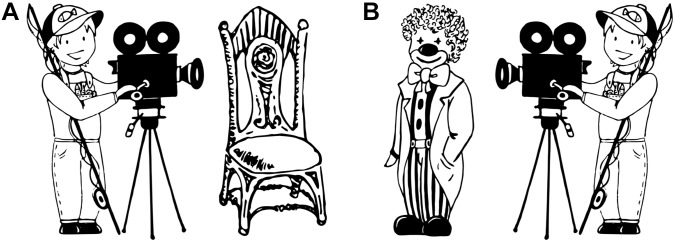
An example of visual stimuli showing **(A)** an agent on the left with an inanimate patient and **(B)** an agent on the right with an animate patient.

A set of 56 black-and-white drawings depicting event scenes between two entities (e.g., a fisherman filming a clown) were used as experimental stimuli. Each event scene included an animate agent (e.g., a fisherman’) on the right- or left-hand side of the drawing together with either an animate (e.g., “a clown”) or an inanimate (e.g., “a chair”) patient on the opposite side (see Figure 1). Each animate agent appeared performing the same action twice, once in a scene with an animate patient and once with an inanimate one. Both agents and patients corresponded to grammatically masculine mono- and disyllabic nouns in German in order to control for the potential influence of morphological or prosodic factors that might obscure the effects of our experimental manipulation. The 14 monosyllabic and the 14 disyllabic animate and inanimate patient nouns chosen for the experiment did not include productive derivations or compounds and did not differ in lemma frequency^1^, *M_animate_* = 158594.50, *M_inanimate_* = 73844.07, *t* (13) = 1.62, *SE* = 52406.51, *p* = 0.130. Drawings of experimental stimuli were made in such a way that agents and patients were comparable in size, visual complexity (i.e., number of details), and distance within which they were situated from each other across items. The portrayed transitive interactions between agents and patients involved no direct contact between them and could be recognized as dynamic actions. The verbs that corresponded to the depicted events were comparable in terms of their likelihood to occur in active and passive voice frames. In addition, two pictures of a red circle subtending an area of approximately 1° of visual angle and centered in the right or the left half of the screen were prepared to realize the cueing of patients.

#### Event Scenes Pre-test

An offline pre-test of experimental stimuli was conducted in order to make sure that participants have similar visual preferences for the depicted scenes with left- and right-positioned agents irrespective of the particular event type. A sample of 36 native speakers of German (33 female, 3 male, mean age 24.2 years, *SD* = 1.8) participated in the pre-test. The pre-test consisted of a questionnaire with nine items corresponding to the following transitive events: *angeln* “to fish,” *filmen* “to film,” *gießen* “to water,” *messen* “to measure,” *schieben* “to push,” *schlagen* “to hit,” *treten* “to kick,” *wiegen* “to weigh,” and *ziehen* “to pull.” Each item contained two mirror images of the same scene and three response options. Participants were asked to mark with a cross the picture they preferred (i.e., the one that – in their opinion – looked more conventional, natural or better) or the option “I have no preference.” No time restriction was applied for completing the questionnaire but participants were instructed to respond as quickly and spontaneously as possible. Two versions of the questionnaire alternated the order in which mirror images for each item were presented. The results showed a significant association between the depicted events and whether or not participants had a preference for left- or right-positioned agents, χ^2^(16) = 26.38, *p* = 0.048. This association was driven by the scene depicting the event *ziehen* “pull,” as significantly fewer participants than expected preferred the left-agent depiction for *ziehen* “pull,” *z* = –2.1, *p* < 0.01; and significantly more participants preferred the right-agent depiction for this verb, *z* = 3.1, *p* < 0.001. When the item depicting *ziehen* “pull” was excluded, participants’ preferences for left- or right-positioned agents were independent of the event type, χ^2^ (14) = 10.84, *p* = 0.699. The verb *ziehen* “pull” was then excluded from the experimental materials, as well as the verb *treten* “kick,” which would require a preposition in the inanimate patient condition.

#### Fillers

A set of 56 drawings of animals and inanimate objects of masculine and feminine grammatical gender that were situated next to or on the top of each other were used as fillers. They served to ensure that participants produced sentences with different syntactic structures (i.e., not involving the description of a transitive event) and did not develop preferences towards a specific sentence type due to repetition from trial to trial.

#### Design

The experimental design included three factors: patient animacy (animate vs. inanimate, within subjects and between items), attention cueing (cue on the patient vs. no cue, within subjects and within items), and patient position (to the right vs. to the left of the agent, within subjects and within items). Four randomized lists presented each item in one of the eight experimental conditions: (1) left-positioned animate patients preceded by a cue; (2) left-positioned animate patients preceded by no cue; (3) left-positioned inanimate patients preceded by a cue; (4) left-positioned inanimate patients preceded by no cue; (5) right-positioned animate patients preceded by a cue; (6) right-positioned animate patients preceded by no cue; (7) right-positioned inanimate patients preceded by a cue; (8) right-positioned inanimate patients preceded by no cue. Each participant was presented with one list and saw items in all 8 conditions, each item appearing in one condition only.

#### Procedure

**FIGURE 2:**
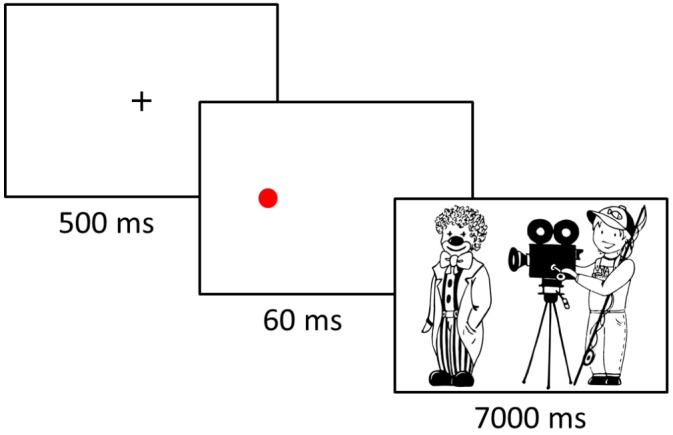
An illustration of the attention cueing paradigm employed in the experiment.

Participants were seated within a 60 cm distance from the computer screen on which the experiment was presented. They were asked to describe scenes on the pictures they would see on the screen in one sentence and were given examples of possible descriptions, as well as several practice trials to make sure they understood the task. The experiment consisted of seven blocks, each block contained eight experimental and eight filler items, which appeared in a random order. Before each block a familiarization phase took place in order to make sure that participants could easily recognize objects and figures that would later appear in the block. During the familiarization phase, objects and figures were displayed individually on the top, bottom, left, and right of the screen and participants had to point at them by using the keyboard keys to answer the questions they heard via headphones, such as “Where is the clown?.” During the experimental phase, participants saw the fixation cross in the middle of the screen (500 ms) and then – depending on the condition (cueing/no cueing) – either a cue placed where a patient would appear next or a blank screen, each for 60 ms. Finally, the scene was presented and participants had 7000 ms to produce its description (Figure 2). To ensure the quality of voice recordings, participants wore a PC-headset Hama “Fire Starter” with a stereo headphone and a boom microphone with a frequency range of 50–5000 Hz. Before the experiment began, the nine-point calibration and validation procedures were performed to ensure the accuracy of eye movement recordings. This procedure was repeated whenever the experimenter detected significant deviations between participants’ gaze and the fixation cross that appeared in each trial. Viewing was binocular but only the dominant eye determined using the Miles test^2^ was tracked. At the end of the experiment participants were asked several questions that aimed at identifying whether they were aware of the presented cue or not. The experiment lasted approximately 45 min.

## Results

### Data Analysis

The obtained behavioral data were analyzed with respect to three measures: the produced utterance type, speech onset times, and eye movements. Statistical analyses were conducted in R (RStudio, 2017) using lme4 package (Bates et al., 2014). Linear mixed-effects modeling using *lmer* function was applied to analyze continuous data (e.g., speech onset times), whereas mixed-effects logistic regression using *glmer* function was applied to binomial data (e.g., probability of first saccades). The optimal data transformation for continuous data was determined using the Box-Cox procedure (Osborne, 2010). The factors Position (right/left patient), Animacy (inanimate/animate patient), Cueing (cued/non-cued patient) were assigned sum-coded contrasts as categorical predictors (e.g., Barr et al., 2013; Levy, 2014). Models included these factors and interactions between them as fixed effects, as well as participants and items as random effects (see Baayen et al., 2008)^3^, Model <- lmer/glmer [DV ∼ Position ^∗^ Animacy ^∗^ Cueing + (1 | participants) + (1 | items)]. Converging models were compared using *ANOVA* function. The results reported below are based on the best-fitting models with the lowest AIC value. The exact random effect structure of selected models is indicated in Tables 1–6.

**Table 1:** Main effects and interactions from the mixed-effects logistic regression model on the probability of passive utterances (Model <- glmer [DV ∼ Position ^∗^ Animacy ^∗^ Cueing + (1 | participants) + (1 | items)]).

	*b*	*SE*	*z*	*p*
Intercept (estimated grand mean)	-8.14649	1.5542	-5.24	<0.001^***^
Animacy	0.51001	0.1313	3.88	<0.001^***^
Position	0.34515	0.1246	2.77	0.006^**^
Cueing	-0.02908	0.1247	-0.23	0.816
Animacy × Position	0.14795	0.1288	1.15	0.251
Animacy × Cueing	-0.03229	0.1255	-0.26	0.797
Position × Cueing	-0.05936	0.1246	-0.48	0.634
Animacy × Position × Cueing	0.06863	0.1270	0.54	0.589

*^∗^p < 0.05, ^∗∗^p < 0.01, ^∗∗∗^p < 0.001.*

**Table 2:** The number of observed passive utterances in cued and non-cued patient conditions when first saccades landed on patients or elsewhere.

	Cueing	No cueing	Total
First saccades to patients	54	40	94
First saccades elsewhere	21	37	58
Total	75	77	152

**Table 3:** Main effects and interactions from the mixed-effects linear regression model on speech onset times (Model <- lmer [DV ∼ Position ^∗^ Animacy ^∗^ Cueing + (1 | participants) + (1 | items)]).

	*b*	*SE*	*t*	*p*
Intercept (estimated grand mean)	0.02547	0.00032	79.88	<0.001^***^
Animacy	-0.00003	0.00007	-0.41	0.679
Position	-0.00017	0.00006	-2.96	0.003^**^
Cueing	0.00007	0.00006	1.14	0.257
Animacy × Position	-0.00001	0.00006	-0.10	0.917
Animacy × Cueing	0.00007	0.00007	0.93	0.351
Position × Cueing	0.00005	0.00006	0.89	0.376
Animacy × Position × Cueing	-0.00004	0.00006	-0.65	0.519

*^∗^p < 0.05, ^∗∗^p < 0.01, ^∗∗∗^p < 0.001.*

**Table 4:** Main effects and interactions from the mixed-effects logistic regression model on the probability of first saccades on patients (Model <- glmer [DV ∼ Position ^∗^ Animacy ^∗^ Cueing + (1 + Animacy + Position + Cueing | participants) + (1 + Animacy + Position | items)]).

	*b*	*SE*	*z*	*p*
Intercept (estimated grand mean)	0.54700	0.2715	2.01	0.044^*^
Animacy	-0.59350	0.2747	-2.16	0.031^*^
Position	-2.13420	0.3764	-5.67	<0.001^***^
Cueing	1.45890	0.2811	5.19	<0.001^***^
Animacy × Position	-0.55570	0.3581	-1.55	0.121
Animacy × Cueing	-0.57700	0.3258	-1.77	0.077
Position × Cueing	-0.5626	0.3401	-1.65	0.098
Animacy × Position × Cueing	0.87000	0.4800	1.81	0.070

*^∗^p < 0.05, ^∗∗^p < 0.01, ^∗∗∗^p < 0.001.*

**Table 5:** Main effects and interactions from the mixed-effects logistic regression model on the percentage of dwell time on patients until speech onset (Model <- glmer [DV ∼ Position ^∗^ Animacy ^∗^ Cueing + (1 + Animacy + Position + Cueing | participants) + (1 + Animacy + Position | items)]).

	*b*	*SE*	*t*	*p*
Intercept (estimated grand mean)	0.2643	0.0167	15.83	<0.001^***^
Animacy	-0.0709	0.0155	-4.58	<0.001^***^
Position	-0.0517	0.0152	-3.41	0.001^***^
Cueing	0.0141	0.0136	1.04	0.301
Animacy × Position	-0.0191	0.0187	-1.02	0.308
Animacy × Cueing	0.0163	0.0199	0.82	0.414
Position × Cueing	-0.0267	0.0176	-1.52	0.130
Animacy × Position × Cueing	0.0320	0.0262	1.22	0.222

*^∗^p < 0.05, ^∗∗^p < 0.01, ^∗∗∗^p < 0.001.*

**Table 6:** Main effects and interactions from the mixed-effects linear regression model on the percentage of total time (full trial) spent on patients (Model <- lmer [DV ∼ Position ^∗^ Animacy ^∗^ Cueing + (1 | participants) + (1 | items)]).

	*b*	*SE*	*t*	*p*
Intercept (estimated grand mean)	0.63650	0.0105	60.65	<0.001^***^
Animacy	-0.06443	0.0116	-5.56	<0.001^***^
Position	-0.00162	0.0107	-0.15	0.880
Cueing	-0.00434	0.0111	-0.39	0.695
Animacy × Position	-0.00930	0.0155	-0.60	0.549
Animacy × Cueing	0.00543	0.0164	0.33	0.741
Position × Cueing	0.00009	0.0152	0.01	0.995
Animacy × Position × Cueing	0.00913	0.0220	0.42	0.678

*^∗^p < 0.05, ^∗∗^p < 0.01, ^∗∗∗^p < 0.001.*

### Utterance Types

The total of 2464 produced utterances can be classified in three structural categories: active sentences (93.43%), passive sentences (6.17%) and other structures (e.g., sentences describing the location of patients, 0.40%). Table 1 shows the results of the mixed-effect model and presents regression estimates (*b*), standard errors (*SE*), *z-*values, and *p*-values for each main effect and interaction. The main effect of the factor position revealed that there were more passive utterances produced to describe scenes where patients appeared on the left of the agent (*M* = 0.07, *SD* = 0.26, *SE* = 0.01) than on the right (*M* = 0.05, *SD* = 0.22, *SE* = 0.01). The main effect of animacy showed that the probability of passive utterances was higher in the descriptions of scenes with animate (*M* = 0.08, *SD* = 0.27, *SE* = 0.01) rather than inanimate patients (*M* = 0.05, *SD* = 0.21, *SE* = 0.01). There was no significant difference in the number of produced passive utterances after cued (*M* = 0.06, *SD* = 0.24, *SE* = 0.01) and non-cued patients (*M* = 0.06, *SD* = 0.23, *SE* = 0.01). At the same time, relating the observed utterance types to eye movement data (see Table 2) revealed a significant association with first saccades to patients (χ^2^ (1) = 18.52, *p* < 0.001). Based on the odds ratio, the odds of producing passive utterances were 2.1 times higher when first saccades landed on patients than when they did not. While more first saccades were made to cued rather than non-cued patients (see Table 4 and corresponding analyses), first saccades were related to the production of passive utterances in both cued and non-cued patient conditions (χ^2^ (1) = 10.52, *p* = 0.001 and χ^2^ (1) = 8.10, *p* = 0.004, respectively). No significant interactions were observed.

### Speech Onset Times

**FIGURE 3:**
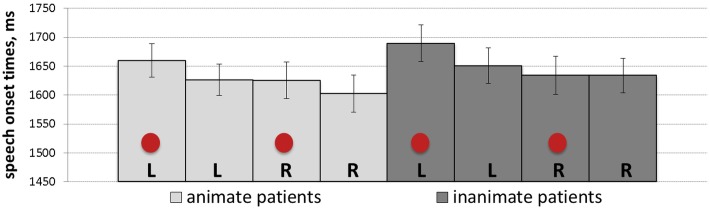
Mean speech onset times (with *SE* as error bars) for each of the experimental conditions: L, R and circles correspond to left-positioned, right-positioned and cued patients, respectively.

Initial stages of data analysis involved identifying the exact time latencies from the onset of the scene picture on the screen until speech onset by using the Praat software (Boersma and Weenink, 2017). Based on the Box-Cox procedure, the reciprocal square root transformation was identified as an optimal transformation and applied to speech onset times. The results are reported for speech onset times of all produced utterances irrespective of the utterance type. Statistical analyses of speech onset times for produced active utterances alone produced the same patterns as described below. Speech onset times of passive utterances could not be analyzed due to the small number of observations. Table 3 shows the results of the mixed-effect model and presents regression estimates (*b*), standard errors (*SE*), *t-*values, and *p*-values for every main effect and interaction. The analyses revealed a significant main effect of patient position, which was due to later speech onset times when patients in scenes appeared to the left of agents (*M* = 1659.96, *SD* = 523.23, *SE* = 14.92) than when they appeared to the right (*M* = 1636.93, *SD* = 587.54, *SE* = 16.75). Mean speech onset times for each of the experimental conditions are shown in Figure 3.

### Eye-Movement Data

**FIGURE 4:**
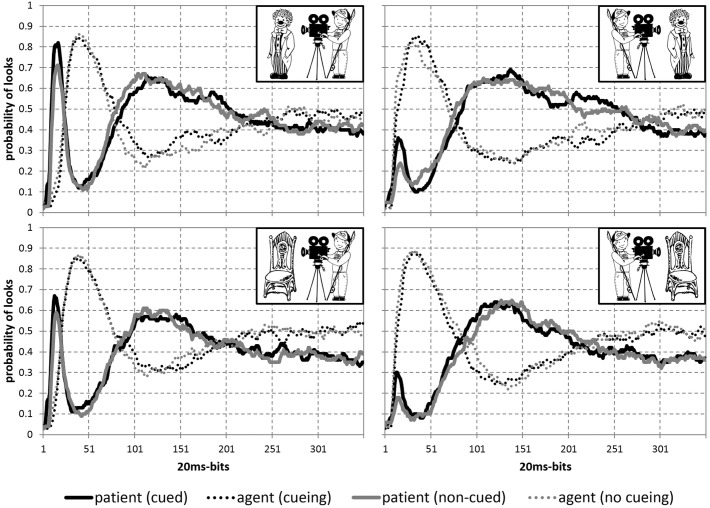
Probability of looks to patients (solid lines) and agents (dotted lines) from the onset of stimuli and until the end of trial (7000 ms) in all 8 conditions: with animate (top) and inanimate (bottom) patients to the left (left) and to the right (right) of agents after patients were cued (black) or not (gray).

The probability of looks in all 8 conditions is presented in Figure 4, which gives an overall impression about eye-movement behavior during scene presentation. Statistical results are reported to reflect the time course from the earliest to later stages, covering the time window before speech onset, as well as the full trial duration. The described measures representing each of these stages include the probability of first saccades, the percentage of dwell time on patients until speech onset and the percentage of total time spent on patients. Other measures reflecting the initial stage in looking behavior (e.g., the percentage, duration and start time of first fixations on patients, the number of first saccades to patients), as well as the time windows until speech onset and offset of stimuli (e.g., the probability of saccades, number of runs, gaze duration), reflect the same eye movement patterns as reported below and therefore are not reported for brevity.

**FIGURE 5:**
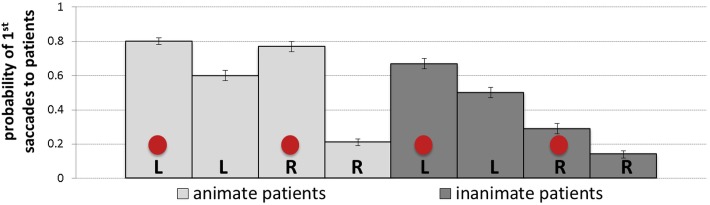
Means (with *SE* as error bars) for probabilities of first saccades to patients in each of the experimental conditions: L, R and circles correspond to left-positioned, right-positioned and cued patients, respectively.

**FIGURE 6:**
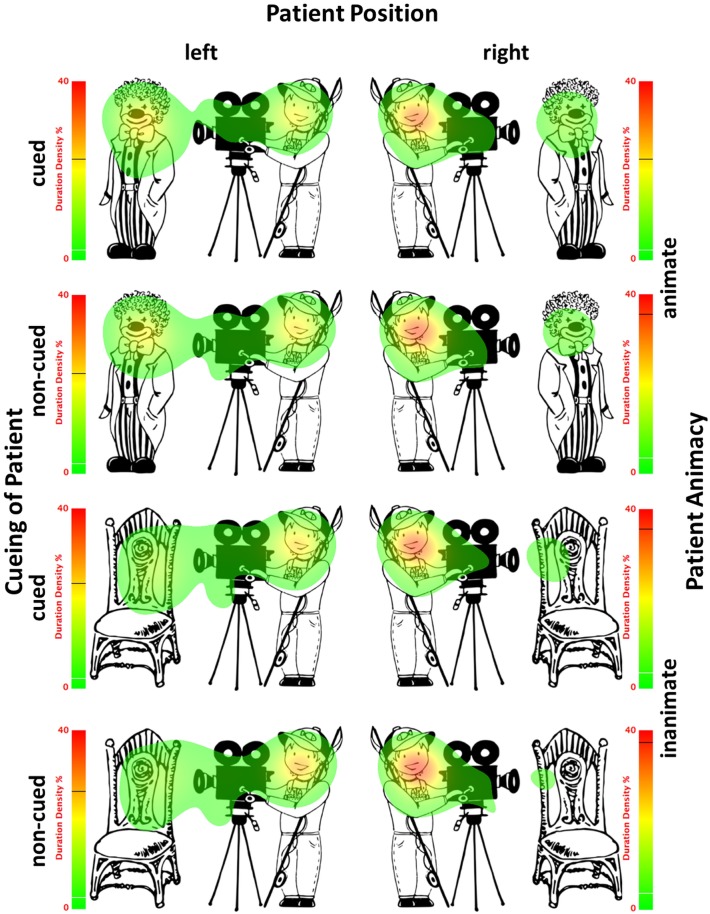
Heatmaps based on duration density show the proportion of fixation duration relative to the trial total (time window: 250–400 ms from the scene onset) for each experimental condition. Black lines on the heatmap scales indicate the actual maximum activation and white lines indicate the 10% activation cut-off.

The probability of first saccades is a measure reflecting the earliest eye movements towards patients. Table 4 presents the results of the mixed-effects model (regression estimates, standard errors, *z*- and *p*-values) on the probability of first saccades landing on patients and reveals significant main effects of each of the manipulated variables. The main effect of animacy was due to the higher probability of first saccades to animate (*M* = 0.49, *SD* = 0.50, *SE* = 0.01) than inanimate patients (*M* = 0.40, *SD* = 0.49, *SE* = 0.01). The main effect of position showed more first saccades to patients after they appeared to the left (*M* = 0.64, *SD* = 0.48, *SE* = 0.01) than to the right of agents (*M* = 0.25, *SD* = 0.44, *SE* = 0.01). The main effect of cueing showed more first saccades to patients after they were cued (*M* = 0.53, *SD* = 0.50, *SE* = 0.01) than when they were not cued (*M* = 0.36, *SD* = 0.48, *SE* = 0.01). Mean probabilities of first saccades to patients for each condition are represented in Figure 5. Heatmaps for each experimental condition visualizing the proportion of fixation duration relative to the trial total within the time window of 250–400 ms from the scene onset are provided in Figure 6.

**FIGURE 7:**
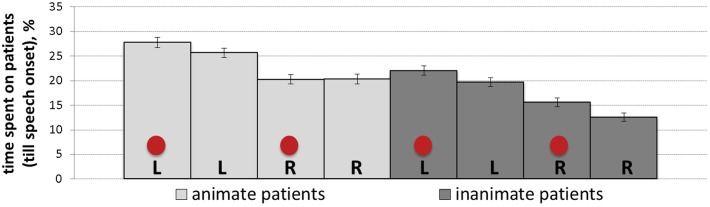
Mean percentage of time (with *SE* as error bars) spent on patients before speech onset in all conditions: L, R and circles correspond to left-positioned, right-positioned and cued patients, respectively.

The percentage of dwell time spent on patients until speech onset reflects gaze behavior from the scene onset until the average speech onset time (1584 ms). Table 5 summarizes the results of the mixed-effects model (regression estimates, standard errors, *t*- and *p*-values) that yielded significant main effects of animacy and position, which were consistent with initial stages of looking behavior. Prior to speech, there was more gazing time spent on animate patients (*M* = 0.24, *SD* = 0.17, *SE* = 0.005) than inanimate ones (*M* = 0.17, *SD* = 0.16, *SE* = 0.004), as well as on left-positioned (*M* = 0.24, *SD* = 0.17, *SE* = 0.005) than right-positioned patients (*M* = 0.25, *SD* = 0.44, *SE* = 0.012). Figure 7 shows mean percentage of gaze time on patients for each experimental condition.

**FIGURE 8:**
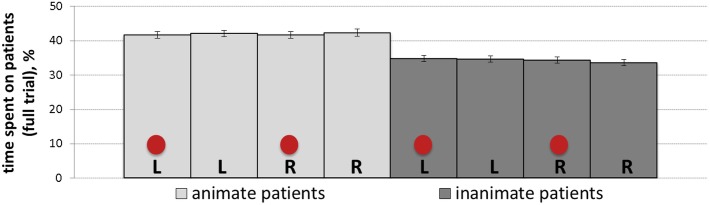
Mean percentage of total time (with *SE* as error bars) spent on patients in all conditions: L, R and circles correspond to left-positioned, right-positioned and cued patients, respectively.

The percentage of total time spent on patients is a measure that represents the time spent on patients throughout the full trial irrespective of speech onsets. Table 6 summarizes the results of the mixed-effects model (regression estimates, standard errors, *z*- and *p*-values) after the square root transformation. The analyses yielded a main effect of animacy showing significantly more gaze time on animate (*M* = 0.42, *SD* = 0.17, *SE* = 0.01) compared to inanimate patients (*M* = 0.34, *SD* = 0.16, *SE* = 0.01). Figure 8 represents mean percentage of total time spent on patients in all conditions.

## Discussion

Unlike previous studies, that either focused on referent animacy *or* referential cueing, here we examined both factors in one sentence-production experiment. Thus, for the first time, these rather diverse factors were considered within one experimental design rather than in isolation. In particular, the present study examined to which extent visual (cueing and patient position) and conceptual (patient animacy) factors lead to systematic variations in the syntactic choice speakers make in describing event scenes, in speech onset times of produced descriptions, and in eye-movement patterns. As to the syntactic choice, speakers were more likely to place patients into the prominent subject position producing passive voice descriptions for scenes when patients were animate or positioned to the left of the agent. Moreover, the production of passive voice descriptions was higher when speakers first looked at patients, irrespective of whether it was due to cueing or not. Speech onset times were also influenced by patient locations and reflected additional costs for scenes where patients appeared to the left of agents. At the same time, speech onset times remained unaffected by either cueing or animacy of patients in the scenes. In contrast to both syntactic choice and speech onset times, all of the manipulated factors had an immediate effect on speakers’ eye movements, so that participants were more likely to look at cued, animate, and left-positioned patients than to their counterparts. Whereas cueing had an impact on the initial looks toward the patient, it did not influence later eye movements. Similarly, the position of patients affected the looks to patients until the initiation of speech but not later on. In fact, the animacy of patients was the only factor that modified both earliest and later eye-movement patterns, such that animate patients were looked at more than inanimate ones.

To summarize, visual and conceptual properties of scenes influenced different aspects of behavior affecting both language and eye-movement responses. In comparison to cueing that only affected eye movements, both patient animacy and position also modified language behavior. While the impact of referent position has been demonstrated in a variety of comprehension tasks, we provide the first evidence that this factor also impinges on sentence production. We will now discuss the effects of each of these factors individually and then turn to their time course relative to one another.

### Patient Animacy

Voice selection was sensitive to the animacy of thematic roles, so that more passive utterances were produced for scenes where the patient was animate. Since passive voice structures in German require placing a thematic patient into a sentence-initial subject position, this means that animate patients were more likely to be verbalized as subjects and to occur at the beginning of an utterance than inanimate patients. This finding is in line with reported animate-first effects in language production and comprehension (e.g., McDonald et al., 1993; MacDonald et al., 1994; Trueswell et al., 1993; van Nice and Dietrich, 2003; Bornkessel-Schlesewsky and Schlesewsky, 2009; van de Velde et al., 2014). These effects are generally attributed to two separate processes that occur during utterance production. The first one relies on word order and consists in placing animate entities in the prominent position at the beginning of the utterance. The second one concerns the assignment of grammatical functions, so that animates are assigned subject functions. It cannot be determined from our data whether these processes occur in two separate stages – with animacy first determining the function and then the position of arguments (Bock and Levelt, 1994) – or simultaneously, as some would argue (e.g., Branigan et al., 2008).

Interestingly, although both object topicalizations (i.e., fronting the object, as in *Den*_ACC_ *Angler filmt der*_NOM_ *Clown* “The fisherman_ACC_ is filming the_NOM_ clown”) and passivizations are grammatically equally valid options in German, no topicalizations were produced in our experiment. The complete absence of OS topicalizations among utterances observed in our experiment may seem puzzling, especially given that these constructions are reported to make up just under 4% of all sentences in German corpora (3.7% – Hoberg, 1981; 3.3% – Kempen and Harbusch, 2005) and were successfully elicited in previous studies with sentence production tasks (e.g., Myachykov and Tomlin, 2008). This incongruity, however, may be explained if the ratios for specific object cases are considered: depending on the corpus, object topicalization with accusative objects only amount to 0.2–0.5% of all utterances, while the remaining 3.1–3.2% of utterances occur with dative objects (see Bader and Häussler, 2010, for more details on the comparison of corpora in this respect). This bias toward dative objects in object topicalized sentences may account for the lack of their occurrence in our experiment, where only the topicalization of accusative objects was possible. At the same time, the ratio of passive sentence occurrences in our experiment (6.2%) closely corresponds to ratios found in corpus studies for German language (e.g., 7% – Brinker, 1971; 9% – Schoenthal, 1976). Our experimentally elicited utterances, thus, reflect quite accurately naturally occurring syntactic variations in language. Nonetheless, neither of the two accounts – grammatical function or word order – can be completely ruled out to explain animacy effects observed in our findings. Nevertheless, both of these models of language production assume a higher conceptual accessibility of animate referents underlying functional and positional processing and our data confirm this assumption, in that animate referents were both assigned subject roles and placed first in produced passive utterances more often than inanimate ones.

The higher conceptual accessibility of animate referents is often related to the inherent significance of animacy as an ontological category and its multifaceted influence on human cognition, including its prioritizing in language use (e.g., Yamamoto, 1999; Dahl, 2008). The priority of animate over inanimate entities in language is conceptualized as a prominence scale that organizes arguments of a thematic structure in terms of a hierarchy (e.g., Lamers and de Swart, 2012). The prominence hierarchy may map on other hierarchies, for instance, that of syntactic functions (subjects ranking over objects) or thematic roles (agents ranking over patients), and thus influence argument linearization. According to the so-called principle of harmonic alignment (Aissen, 2003), higher-ranked entities on one scale should align with higher-ranked entities on another scale. In case of passive utterances produced in our experiment, it was the animacy hierarchy that aligned with that of syntactic functions, so that more prominent animate patients were given higher-ranked subject functions. On the one hand, this is consistent with theories about the higher prominence of animate versus inanimate entities confirming a bias in the perception of animate roles as fitting subject functions better than inanimate ones. On the other hand, thematic roles are typically reported to align with syntactic functions, so that agent and not patient roles function as sentential subjects (e.g., Dik, 1978; Jackendoff, 1987; Grimshaw, 1990). In this respect, the production of passive utterances in our experiment provides an example of how semantic prominence of animacy may override the prominence of thematic roles.

Taken together, our experiment confirms that animacy is an important conceptual factor that can affect speakers’ structural choices. This finding is in line with previous studies that involved the manipulation of agent animacy. Crucially, we investigated the animacy status of patients, thereby corroborating the importance of animacy for structural choices even when less prominent patient arguments are considered.

### Visual Cueing

In contrast to the manipulation of patient animacy, drawing attention to patients using visual cueing did not elicit expected changes in language production. Nevertheless, the visual behavior was affected as predicted, so that upon scene presentation gaze was first directed to cued rather than non-cued patients. Thus cueing was effective in altering eye movements, even though it had no impact on either the onset of produced utterances or their syntactic structure. Given that the effects of cueing only surfaced in the initial saccades and fixations, it should not be surprising that these short-lived effects did not affect speech production. Yet, this finding contradicts a number of previous studies (e.g., Gleitman et al., 2007; Myachykov et al., 2011, 2012) that did find a correspondence between the increased use of passive voice and the visual cueing of patients in scene description tasks. It is assumed that increasing the saliency of patients via cueing may make them more accessible for processing and therefore more likely to be assigned subject functions, which then results in a passive voice utterance (e.g., Myachykov et al., 2018b). Despite the apparent similarity between these studies and our experiment, however, there are important methodological differences that could be responsible for the discrepancy in results. Thus, cueing manipulation in these aforementioned studies typically consisted in cueing both agents and patients, which perhaps created a starker contrast between the two cueing conditions compared to our experiment where agents were never cued and only patients were either cued or not. Moreover, cueing in these studies targeted the visual salience of referents, whereas their conceptual characteristics (e.g., animacy) did not vary systematically. In our study, the conceptual prominence exerted by animacy also rendered patients more accessible, conceivably outweighing the increase in their saliency due to cueing. As a result, the shifts in visual attention towards patients following cueing in our experiment may be shorter lasting than in previous studies. Unfortunately, this cannot be determined, as analyses of eye movements reflecting the time course of changes in visual attention were not reported in these studies.

While differences in the employed paradigms may have contributed to the absence of an effect of visual cueing on sentence production, other studies have likewise failed to observe effects of attentional cueing on structural choice (e.g., Myachykov et al., 2011; van de Velde et al., 2014; Hwang and Kaiser, 2015). For instance, Myachykov et al. (2011) did not observe significant effects of visual cueing on Finnish speakers’ structural choice in a picture description task despite the fact that the visual cue effectively shifted participants’ gaze to the cued entity. The same was true for speakers of Dutch (van de Velde et al., 2014) as well as for speakers of Korean (Hwang and Kaiser, 2015). A number of reasons could account for discrepancies in the results when it comes to cueing affecting (or not) speakers’ structural choices. One reason might be the cross-linguistic variability in the grammatical systems of different languages. In a language with a case system (like German), for instance, the accessibility of a patient increased by cueing may be interfered with by the necessity to provide a case marked article (*den* “the_ACC/MASC_” or der “the_NOM/MASC_”) before the noun. The choice of the case marking on the article determines the choice of syntactic structure. If the accusative case marked article *den* has been chosen, only an object topicalization can follow (*Den*_ACC_ *Angler filmt der*_NOM_ *Clown* “The fisherman_ACC_ is filming the_NOM_ clown”). In contrast, the choice of the nominative article *der* necessitates to procede with a passive (*Der*_NOM_ *Angler wird vom*_DAT_ *Clown gefilmt* “The fisherman is filmed by the Clown”). As we have seen in corpora data described above, accusative objects are almost never topicalized, suggesting that the speaker would rather recur to the passive voice in order to produce a grammatically acceptable utterance. A related reason would be the relative flexibility of word order in German as compared to English. Since the number of available structural options is higher in a language with flexible word order, it may be more challenging for speakers of that language to integrate their linguistic choice with the shifts of visual attention. However, it is also possible that using longer cues may help increase the accessibility of referents enough to overcome language-specific factors that may interfere with structural choices. Cue duration does seem to play a role for speakers of English (Myachykov et al., 2018a). However, its role for speakers of other languages remains to be clarified in future studies. In sum, our findings indicate that increasing visual salience of referents by means of visual cueing may not be as effective in influencing the utterance structure as previously reported.

### Spatial Position of Patients

Although it has often been observed that the position of referents is affected during sentence comprehension (e.g., Maass and Russo, 2003; Dobel et al., 2007), so far no study has looked at this effect in language production. However, our results show that the positioning of patients in space had a pervasive influence on participants’ behavior affecting early and later eye movements, as well as the initiation of utterances and voice selection. The effects of patient positioning across all of these behavioral measures were consistent with our predictions and revealed participants’ bias to expect agents to the left of patients in visual scenes and to assign subject functions to left-positioned rather than right-positioned referents. Similar spatial biases have been documented for areas other than sentence production. There is converging evidence that the relatedness of spatial positioning of referents and their thematic roles becomes evident in language comprehension. Dobel et al. (2007), for instance, asked participants to either draw or arrange transparencies of protagonists or objects in order to depict sentences they heard. Their findings suggest that the leftmost position in space is associated with agents rather than patients. Similar findings come from experiments by Chatterjee et al. (1995, 1999), where the recognition of agents was less effortful when agents appeared to the left than to the right of recipients. Moreover, the applied spatial schema seem to also affect the direction in which the action evolves, i.e., from left to right (Chatterjee, 2002). In line with these findings, our results confirm a similar left-agent bias for sentence production. Crucially, this effect appears to be modulated by writing direction, as the reverse bias is found in speakers of languages with left-to-right scripts (e.g., Maass and Russo, 2003). For instance, a recent study that investigated spatial preferences for agent placement in scenes depicting transitive actions suggests its dependence on script direction (Esaulova et al., unpublished). The authors evaluated visual preferences for left- and right-positioned agents in a group of native German and a group of native Arabic speakers. The results showed that speakers’ visual preferences were consistent with the script direction in their native languages: German speakers preferred pictures with left-positioned agents, while Arabic speakers preferred those with right-positioned agents. In addition to language-related effects of visual positioning of referents, left-to-right spatial schemata have also been observed in a number of other areas that nevertheless correlate with script direction (Zebian, 2005; Santiago et al., 2007; Pérez et al., 2011). One example of such a spatial bias is the so-called SNARC effect – a tendency to envisage numbers and magnitude along a horizontal line, starting with the smallest item and moving to the largest from left to right (Hubbard et al., 2009). Again, this tendency occurs in languages with a left-to-right script, while the opposite right-to-left pattern – known as the Reverse SNARC effect – is observed for languages with a right-to-left script, such as Hebrew or Arabic (e.g., Zebian, 2005; Shaki et al., 2009). Just like SNARC and time representations, schemata for linguistic agency change their direction in populations with right-to-left script (e.g., Arabic – Maass and Russo, 2003; Esaulova et al., unpublished). Furthermore, the spatial mental schemata seem to be used to represent social psychological concepts, such as social agency (see Suitner and Maass, 2011, for an overview on Spatial Agency Bias). Higher-status social groups (e.g., men) are typically mentioned before lower status groups (e.g., women) and are therefore positioned to the left in left-to-right languages (Hegarty et al., 2011). Likewise, groups that are represented to the left are generally perceived as the “norm” and of higher status (Hegarty et al., 2010; Bruckmüller et al., 2012). Patients positioned on the left in event scenes in our experiment could be perceived as more agentic than those on the right facilitating both the assignment of subject functions to them and a word order in which they would be mentioned first. Thus, while spatial orientation of patients may appear as a merely visual factor, it could in fact reflect both visual and conceptual preferences. Moreover, it can be conceptualized as a prominence-lending factor, since – similar to animacy – it can be represented as a hierarchy with left-positioned referents aligning more readily with subjects than right-positioned ones.

Our findings suggest that positioning of figures and objects in event scenes influences sentence production in two ways, affecting both the structure and onset times of produced utterances. The position of agents and patients relative to one another is thus not only relevant for language comprehension, as previously reported, but also for language production. Whether these effects in language production may be subject to cultural adaptation – as it appears to be the case in language comprehension – is yet to be determined. In any case, considering that counterbalancing seems to be a common practice in most studies examining sentence production during scene descriptions (e.g., Myachykov et al., 2018a), our finding has important methodological implications. Since our experiment demonstrates that the positioning of referents exerts a strong effect on sentence production, counterbalancing may not be adequate or sufficient to account for its influences. Instead, the materials should be controlled more carefully and/or the effects of positioning should be systematically explored and reported.

### The Time Course and Interplay of Visual and Conceptual Influences

Our study addressed visual and conceptual factors within the same experiment targeting visual and language responses, which allowed us to explore the time course of influences related to each of these factors, as well as whether they interact and if so, on which behavioral level.

The time course of influences exerted by each of the manipulated factors appears to depend on their relatedness to meaning. Patient animacy as a conceptual property drawing on meaning did not only have immediate but also long-lasting effects shaping both the initial and later visual inspection of scenes, as well as syntactic choices for their description. The impact of animacy is therefore both early and long lasting. This observation is in line with the findings of an EEG study by Malaia and Newman (2015) who investigated the syntax-semantics interface during word-by-word reading of sentences where subject animacy and verb telicity were varied (e.g., *The witness*_animate_*/mansion*_inanimate_ *seized*_telic_*/protected*_atelic_* by the agent was in danger*). The authors found neural support for first-noun animacy affecting the online comprehension of not only that noun but also later parts of the sentence, demonstrating that animacy effects persisted during sentence comprehension. In contrast to the long-lasting effects of animacy, our findings revealed that visual cueing which was unrelated to any conceptual interpretation only impacted initial scene scanning patterns but no later changes in gaze or speech. The effect of visual cueing can thus be considered early but rather short-lived. At the same time, another visual factor – the spatial position of patients – seems to have an intermediate effect, as it influenced the immediate gaze behavior, as well as syntactic choices and utterance initiation times. The impact of conceptual properties can thus be seen from the very onset of a visual scene until after the description is produced, while the influence of visual properties drawing on perceptual mechanisms reduces over time – be it immediately upon the onset of the scene (cueing) or once the syntactic choice is made (patient position).

Interestingly, the position of patients had an impact similar to that of animacy in that it did not only affect eye movements until speech onset but also influenced speaker’s structural choices leading to more passivizations when patients were located to the left of agents. Moreover, patient position did not only affect the type of produced utterance but also their onsets, leading to longer delays in case of left-positioned patients. This could be related to a left-to-right bias we described earlier, as well as to a misalignment between the incrementally planned and structurally unmarked agent-first structure and the visual input. While both animacy and position effects emerged very early as judged by changes in gaze, the effect of patient position was relatively short-lived compared to that of patient animacy. In this sense, the spatial organization of thematic roles in a scene may also be given an additional meaning (i.e., of agency). This conceptual interpretation of an otherwise visual factor goes beyond its visual properties and confers it an intermediate status compared to visual cueing on one side and conceptual animacy on the other.

Although different factors indeed affected different aspects of sentence production and looking behavior, none of them interacted. This could mean that rather than depending on or interfering with each other, visual and conceptual factors exert their influences independently. Along with this, the relative importance appears to differ from factor to factor. In this way, the conceptual factor of animacy seems to be more powerful in making entities prominent than changes in visual saliency due to cueing. This is in line with Henderson et al. (2018), who argued that conceptual aspects affect the perception of visual scenes more than visual ones. At the same time, however, this finding is at odds with Rissman et al. (2018) who suggest that visual saliency may override conceptual saliency related to animacy. It is worth noting, however, that disentangling with certainty conceptual and visual saliency of animate vs. inanimate entities in depictions is highly problematic, since some of the characteristics (e.g., a possession of a face) that are intrinsic animate features (i.e., conceptual) may also increase their visual saliency. Whether animacy may be overridden by visual cues should be confronted in future research, which would vary the saliency of visual cues by manipulating such parameters as cue duration or size. So far, however, our findings suggest that when a scene combines both conceptual information (i.e., animacy) and visually salient features (i.e., cue), features loaded with meaning outrank perceptual saliency.

## Conclusion

Unlike previous experiments, our study investigated the influence of visual and conceptual properties of scenes together rather than individually. We also considered a number of behavioral responses (gaze changes, speech onset, utterance structures) aiming at a fuller picture, as opposed to studies that targeted either speech or visual behavior. Applying this approach, we were able to demonstrate – for the first time – that spatial positioning of patients in scenes manifests in language production. Moreover, our experiment suggests that the position of referents in an event scene may increase their prominence similar to animacy. This is reflected in speakers’ tendencies to assign left-positioned referents subject functions and to place them in initial sentence positions. Importantly, structural choices were affected by the manipulation of patients’ and not agents’ animacy status indicating that features like animacy may increase the prominence of both agent and patient roles in a sentence. The relative weight of visual cueing and patient position appears to gradually reduce over time, while conceptual factors drawing on meaning have longer-lasting effects. Therefore, increasing visual salience of referents by means of visual cueing may not be as effective in influencing sentence production as previously reported. Future studies are needed to clarify this discrepancy in findings by considering if visual factors (e.g., duration or type of cue) and/or language-specific characteristics may be possible reasons for such differences.

## Ethics Statement

This study was carried out in accordance with the recommendations of the Ethics Commission of Cologne University’s Faculty of Medicine with written informed consent from all subjects. All subjects gave written informed consent in accordance with the Declaration of Helsinki. The protocol was approved by the Ethics Commission of Cologne University’s Faculty of Medicine.

## Author Contributions

YE designed the study, collected and analyzed the data, and wrote the manuscript. MP and SD designed the study, and wrote the manuscript.

## Conflict of Interest Statement

The authors declare that the research was conducted in the absence of any commercial or financial relationships that could be construed as a potential conflict of interest.

## Footnotes

1 Frequency analyses were based on collected frequencies of word forms including inflections using the corpora from the Archive of written language, Institute for German Language, Mannheim, Germany.

2 Participants were asked to fixate a point on the wall through a small opening created by their hands with arms extended. Then they drew hands closer to face while fixating the point. Depending on the ocular dominance, they moved hands to the left or the right side of the face, in order to keep focusing on the point with the dominant eye.

3 Trial order (centered) was initially included as a covariate but did not influence the results and was removed to simplify the model structure.
